# The COVID-19 Pandemic Exposes Another Commercial Determinant of Health: The Global Firearm Industry

**DOI:** 10.9745/GHSP-D-20-00628

**Published:** 2021-06-30

**Authors:** Adnan A. Hyder, Meghan Werbick, Lauren Scannelli, Nino Paichadze

**Affiliations:** a George Washington University, Milken Institute School of Public Health, Washington, DC, USA.

## Abstract

Firearm violence is a public health crisis worsened by lobbying, marketing, and supply chain tactics from the private industry. During the heightened burden of the COVID-19 pandemic, public health practitioners should use a commercial determinant of health lens to combat this threat.

## Key Messages

- Firearms have a large impact on the health of individuals and societies globally, with a disproportionate burden on low- and middle-income countries (LMICs).
- The firearms industry uses strategies to promote the sale and use of their products that are detrimental to health and therefore should be viewed through a commercial determinants of health lens.
- Coupled with the heightened risks during the COVID-19 pandemic, the threat to health posed by the firearms industry necessitates public health research, intervention, and collaboration.
- Public health practitioners and policy makers should increase efforts to reduce the burden of firearm violence.
- Public health researchers should use a commercial determinants of health lens when investigating health risks caused by firearms.
- When discussing solutions to firearm violence, public health practitioners and policy makers should include perspectives from LMICs and vulnerable groups.

## INTRODUCTION

As a global public health community, we are constantly confronting new attacks on our health and safety. Public health officials have been forced to reassess how to address, research, and fight threats to our health in the face of a changing environment. One of these persisting threats is firearm violence. Firearms contribute to more than 250,000 recorded deaths each year worldwide and 230 per 100,000 years of life lost; these numbers suffer from potentially serious underreporting.^1^ Unfortunately, low- and middle-income countries (LMICs) suffer a disproportionate burden of firearm violence. Research has shown that 83% of all violence-related deaths occur in LMICs.^2^ Moreover, in the United States, 90% of the burden of firearm violence falls on civilian populations, as compared to the 10% concentrated in armed conflict situations, and the societal costs of firearm violence have reached more than $150 billion annually.^1^ As a private industry, gun producers and distributors play a major role in the growing availability of guns and, in turn, the severity of firearm violence in the U.S. and globally. We discuss some of these problems here and issue a call to action for the public health, medical, and social communities.

## PRIVATE INDUSTRY'S ROLE IN FIREARM VIOLENCE

Commercial determinants of health are defined as^3^:

*strategies and approaches used by the private sector to promote products and choices that are detrimental to health.*

Private-sector industries, such as alcohol, tobacco, food, beverages, pharmaceuticals, and automobiles, frequently rely on marketing, lobbying, corporate responsibility strategies, and extensive supply chains to direct focus away from the diseases that stem from their industries.^3^

The firearms market is no different. In 2017, more than 1 billion firearms were in circulation globally, 85% of which were in civilian hands.^4^ Of those, roughly 46% or 393 million were in 1 country—the United States—where the staggering number of guns now exceeds the population. For decades, the firearm industry has worked to minimize the truth about firearm violence through marketing and lobbying, similar to tactics used by the alcohol and tobacco industries.^5^ As a result, public health advocates have worked hard to expose the true impact of firearms on health and society.

The influential role of the private firearm industry on public perspectives holds true in LMICs. Brazil—the sixth leading country in firearm deaths per 100,000 people—is the second-largest producer of weapons in the western hemisphere. Increases in Brazil's domestically produced weapons have correlated with increases in firearm violence.^6^^,^^7^ Despite these trends, the firearm industry in Brazil argues that the increase in violence is due to “imported” small arms to influence policy decisions and continues with their local production.^7^ As the United States is also a large importer of weapons from Brazil, their extensive supply chains ensure that production and profits in Brazil remain high. Similarly, in Colombia, ongoing domestic conflicts have supported an increased production in small arms, as well as increased imports from the United States.^8^

Unfortunately, the firearms industry is uniquely protected by governmental policies in some countries. For example, the United States firearm industry uses the Second Amendment, a law that protects the right of people to keep and bear arms, as a rallying cry for weakening federal firearm regulations and attacking state regulations. Policies like the Gun Control Act of 1968 and its subsequent amendments aim to reduce the harmful effects of firearms by regulating interstate commerce, requiring background checks on purchasers, and prohibiting certain categories of individuals from purchasing firearms such as minors, mentally ill individuals, and individuals with a criminal record. However, firearm advocacy groups help pave the way for loopholes to circumvent these restrictions. For instance, advocacy groups, such as the National Rifle Association (NRA), have pushed for the inclusion of the “gun show loophole” which exempts private sellers from requiring background checks for purchasers.^9^ The NRA is at the forefront of protecting the firearm industry and one of the most successful advocacy groups. While they claim to promote hunter safety and offer training to members of law enforcement, for the past 30 years a vast majority of their $250 million budget was reportedly used toward limiting gun legislation regardless of the societal impact.^10^ In the past 20 years, of all legislation introduced federally in the United States, only 1 passed into law despite support for gun regulation from the majority of Americans.

Research also suggests that competition within the firearms market has made weapons more lethal over the years.^11^ Increases in foreign imports globally have encouraged manufacturers to make higher-performing weapons, and standardization has allowed most models of small arms to fire any type of ammunition. In some cases, the industry has even increased the lethality of weapons for state-employed small arms production.^11^ We are now living in a society where more people have access to more deadly technology with little training or societal accountability.

It is for these reasons that using a commercial determinants of health lens benefits public health efforts to address and control firearms. We propose that it is important to analyze the firearms industry from a commercial determinants of health perspective to help reduce the burden of firearm violence globally.^12^

## COVID-19 PANDEMIC AND FIREARM VIOLENCE

The COVID-19 pandemic has exasperated many societal issues, firearm violence included. At the onset of the pandemic in March 2020, 1.9 million guns were sold in the United States, marking the second-largest month of sales in history; trailing only the month following the Sandy Hook Elementary School shooting and President Obama's re-election in 2012.^13^ According to the U.S. Federal Bureau of Investigation, nationwide background checks for individuals purchasing firearms in March 2020 were up by 41% from the same time last year.^13^ Exports of firearms to LMICs have also surged during the pandemic. U.S. manufacturers exported more than US$90 million worth of firearms to LMICs—most notably India and Thailand—within the first 5 months of the pandemic in 2020, equal to almost 3 times the number from March to July of 2019.^14^

Exports of firearms to LMICs have surged during the pandemic. U.S. manufacturers exported more than US$90 million worth of firearms to LMICs.

With the subsequent “stay at home” orders in many countries and unprecedented access to such industries online, the COVID-19 pandemic is an increasingly dangerous time for victims of gun injuries including domestic violence. The United States saw a large spike in reports of domestic violence since the onset of the pandemic, and many domestic violence victims are forced to stay in these high-risk domestic situations with little to no support due to lockdown measures.^13^ These increases in gun purchases pose a further threat to victims of domestic violence in the United States and globally. For example, in Bangladesh, more than 50% of women who were already experiencing domestic violence reported increases following lockdowns; while in India, lockdowns led to a 131% increase in domestic violence complaints, with higher rates in areas with stricter measures than those with the least strict measures.^15^^,^^16^

At the same time, people have been forced to deal with increased stresses from the pandemic. Access to mental health services has decreased due to office closures, and studies have shown that 47% of individuals reported experiencing negative mental health effects due to isolation measures, job loss, and worries regarding themselves or a high-risk loved one contracting COVID-19.^13^ The risk of suicide is known to be highest in the first 6 weeks after a person purchases a gun, creating an entirely new burden on people buying firearms and facing stay-at-home orders and stresses related to the pandemic.^13^

## ADDRESSING THE FIREARM VIOLENCE PANDEMIC

During the COVID-19 pandemic, it is important that the field of public health increases efforts to reduce the burden of firearm violence. The commercial determinants of health framework plays a useful role in such an effort by focusing on the industry, flow of arms, and tactics used by distributors, not just users. By further studying this issue, addressing concepts of ownership, industry specifics, concentration, and leverage within the global gun market, we will be able to better understand which aspects of the commercial market have the largest impact on health risks caused by firearms.^17^

To do so, we need a multidisciplinary effort within a commercial determinants of health framework that is inclusive of direct and indirect health outcomes, understanding the political economy of guns and international equity issues (Table). Considerable work has been done over the past 3 decades to document the impact of firearms on health, the economy, marginalized populations, and rates of violence. However, there is a need to further study associated markets, industry tactics globally, and lobbying efforts that increase the potency of firearms as a negative public health issue around the world. The firearm industry and its supporters (industry groups, lobbyists, and paid research groups) especially need to be the focus of global health research, including a study of their transnational practices and marketing strategies. We recommend that future research on firearms uses the proposed framework to address the commercial determinants of health within firearm violence.

To address this epidemic, it is also critical to bring perspectives from LMICs together with countries like the United States and to include populations most affected by firearms, such as those in poverty, women, youth, and minorities. Finally, it is important to bring the best science to bear on this global perspective to enable evidence-based advocacy for national and international audiences and to change mindsets. Using the commercial determinants of health framework will require such perspectives, and by doing so, the field of public health can play its part in reducing the burden of firearm violence worldwide.

**TABLE.** Initial List of Recommendations for Addressing Firearms as a Commercial Determinant of Health

Element of Commercial Determinant of Health	Relevance to Firearm Industry	Recommended Action or Policy
Lobbying	Influencing gun control and gun safety policies	Laws requiring universal background checks and permit-to-purchase lawsGlobal health organizations (e.g., United Nations-World Health Organization) promoting international conventions
Marketing	Targeted marketing to vulnerable groups such as women and the young	Laws restricting marketing and advertising to vulnerable groupsResearch on optimal pathways for affected groups to impact policy change
Corporate Social Responsibility	Emphasis on safety education, training, responsible firearms ownership programs, shifting focus away from commercial sales^18^	Research assessing the negative effects of corporate social responsibility, policies defining corporate social responsibility strategies and implementing regulationsResearch exposing how corporate social responsibility is used by industry to its benefit
Extensive Supply Chains	Large export markets to low- and middle-income countries	Regulating trade and enhancing international export control rules around products harmful to healthResearch on the role of intermediary companies on gun access

## Notes

**Peer Reviewed**

**First published online:** April 29, 2021.
